# Numerical analysis of a second-grade fuzzy hybrid nanofluid flow and heat transfer over a permeable stretching/shrinking sheet

**DOI:** 10.1038/s41598-022-05393-7

**Published:** 2022-01-31

**Authors:** Muhammad Nadeem, Imran Siddique, Jan Awrejcewicz, Muhammad Bilal

**Affiliations:** 1 Department of Mathematics, University of Management and Technology, Lahore, 54770 Pakistan; 2 Department of Automation, Biomechanics and Mechatronics, Lodz University of Technology, 1/15 Stefanowskiego St., 90-924 Lodz, Poland; 3 Department of Mathematics, University of Lahore Gujrat Campus, Gujrat, Pakistan

**Keywords:** Engineering, Mathematics and computing

## Abstract

In this work, the heat transfer features and stagnation point flow of Magnetohydrodynamics (MHD) hybrid second-grade nanofluid through a convectively heated permeable shrinking/stretching sheet is reported. The purpose of the present investigation is to consider hybrid nanofluids comprising of Alumina $\left( {{\text{Al}}_{{2}} {\text{O}}_{{3}} } \right)$ and Copper $\left( {{\text{Cu}}} \right)$ nanoparticles within the Sodium Alginate (SA) as a host fluid for boosting the heat transfer rate. Also, the effects of free convection, viscous dissipation, heat source/sink, and nonlinear thermal radiation are considered. The converted nonlinear coupled fuzzy differential equations (FDEs) with the help of triangular fuzzy numbers (TFNs) are solved using the numerical scheme bvp4c. The numerical results are acquired for various engineering parameters to study the Nusselt number, skin friction coefficient, velocity, and temperature distribution through figures and tables. For the validation, the current numerical results were found to be good as compared to existing results in limiting cases. It is also inspected by this work that with the enhancement of the volume fraction of nanoparticles, the heat transfer rate also increases. So, it may be taken as a fuzzy parameter for a better understanding of fuzzy variables. For the comparison, the volume fraction of nanofluids and hybrid nanofluid are said to be TFN [0, 0.1, 0.2]. In the end, we can see that fuzzy triangular membership functions (MFs) have not only helped to overcome the computational cost but also given better accuracy than the existent results. Finding from fuzzy MFs, the performance of hybrid nanofluids is better than nanofluids.

## Introduction

In heat transfer mechanisms, energy-saving is a fundamental problem in different advanced industrial and technological applications. Over many decades, conservative fluids, for example, polymeric solutions, biofluids, glycols, water, tri-ethylene refrigerants, ethylene, oils, and lubricants, are used as heat transfer fluids. They have a limited capability to transfer heat due to their lower thermal conductivity. But, nanofluid, which is the mixture of nano-sized particles of size 1–100 nm and host fluids, have higher thermal conductivity, consequently enhancing the heat transfer rate. Choi and Eastman^1^ firstly had done several experiments to establish this new revolutionary idea. Adding more than one kind of nanoparticles in the host fluid makes them more advantageous, as, in a single fluid, we can have many physical properties according to our needs. In comparison to nano and regular fluids, hybrid nanofluids have better thermo-physical properties and a faster heat transfer rate. The influence of thermal radiation and natural convective flow on a third-grade fuzzy nanofluid flow between two upright surfaces was analyzed numerically by Nadeem et al.^2^. Siddique et al.^3^ studied the heat transfer and Couette flow on a third-grade fuzzy nanofluid under a fuzzy environment across an inclined plane. The Powell–Eyring hybrid nanofluid flow, convective heat transfer, and the generation of volumetric entropy on a radially horizontal permeable stretching surface were presented by Aziz et al.^4^. Cattaneo-Christov influences on Carreau nanofluid were investigated by Farooq et al.^5^. Several articles on hybrid nanofluids, owing to their importance and usefulness, can be seen in the literature^6–10^.

MHD is the study of the combination of electromagnetism and fluid mechanics, i.e., the behavior of magnetic field on electrically conducting fluid which can control the rate of heat transfer, and flow in a system. Currently, the study of MHD flow has attracted the attention of a large number of scientists due to its significance in numerous industrial and engineering practical applications, such as magnetic mixers, nuclear reactors, chemical reactions, plasma flows, MHD power generators, petroleum industries, metal casting, metallurgical processes and boundary layer control in aerodynamics. Alfven^11^ was the one who coined the term MHD. Nadeem et al.^12^ used the triangle MF to address the uncertainty in MHD and ohmic heating on a third-grade fluid in an inclined channel in a fuzzy environment. In the presence of heat production, thermal radiation, and nanoparticle structure, Saqib et al.^13^ examined the MHD flow of a hybrid Ferro-nanofluid. The hybrid nanofluid effect on MHD boundary layer flow for viscous fluids was studied by Gul et al.^14^. Refs.^15–19^ can provide further information regarding the MHD flow studies.

The well-known fact that many engineers and researchers are attracted to study the non-Newtonian fluids because of their practical applications in many industrial and engineering units such as chemical industry, lubrication, plastics processing, practical biomedical applications, and mining industry, etc. In our study, the fluid model used for the heat transfer and flow purpose is a sub-category of non-Newtonian fluid named a second-grade fluid. This fluid model describes the shear thickening and shear thinning effects and normal stress effects for the steady flow. The thermodynamic study of second-grade fluid was achieved by Fosdick and Rajagopal^20^ and Dunn and Rajagopal^21^. Haq et al.^22^ studied the MHD and Darcy’s law effects on the second-grade fluid flow through a vertical infinite flat plate using Laplace transform (LT). Vajravelu and Rollins^23^ inspected the MHD flow of a second-grade liquid over a stretching surface. Also, the flow of a second-grade fluid over a stretching sheet was deliberated by Vajravelu and Roper^24^. Hayat et al.^25^ scrutinized the flow behaviours of thermophoresis, MHD, and convective heat transfer of second-grade fluid over an exponentially stretching sheet. In another article, Hayat et al.^26^ examined the flow properties of second-grade nanofluid through a non-linear stretching sheet. The heat transfer, mixed convective and thermal radiation flow of second-grade fluid over an exponentially stretching sheet analyzed by Ramzan et al.^27^. Khan and Rahman^28^ studied the modified second-grade fluid flow over a nonlinear isothermal stretching sheet. Imtiaz et al.^29^ scrutinized the magnetic field effect, chemical reaction, and thermal radiation of a second-grade fluid over a curved surface. Zuhra et al.^30^ considered the effect of heat transfer and boundary layer flow of a second-grade fluid over a stretching sheet through the porous medium. The effect of MHD and heat transfer flow of a second-grade hybrid nanofluid flow through an absorbent shrinking/stretching sheet was investigated by Roy and Pop^31^.

In dynamical systems, different kind of fuzziness or uncertainties happens, related to the measurement of errors, material properties, environmental factors, incomplete knowledge, dimensional tolerances, comparison, engineering parameters, initial and boundary conditions, etc. This fuzziness or uncertainties will undoubtedly affect the dynamic systems, and this might change the result because of the dynamic responses. In fluid dynamics, the engineering parameters and the heat transfer parameters like the volume fraction of nanoparticles exist in the governing equations. These are neither measured exactly nor their specific nominal values. So, in actual practice, these values are fuzzy or uncertain because their given information is incomplete, vague, or imprecise. In this situation, fuzzy sets theory (FST) is a useful tool for the phenomena under consideration, and it is more accurate than assuming physical difficulties. For more precisely, the FDEs play a significant role in reducing the uncertainty and proper way to describe the physical problem which arises in uncertain heat transfer parameters, initial and boundary conditions. In 1965, Zadeh^32^ had given an alternate idea of set theory which is named FST, and this approach handles the imprecise or uncertain information. The notion of fuzzy number (FN) was presented by Chang and Zadeh^33^. Further, these numbers were generalized by Dubois and Prade^34^. Different types of FNs can be categories in triangular, trapezoidal, and Gaussian fuzzy numbers. Here we consider TFNs for the sake of completeness. In 1987 Seikkala^35^ introduced the concept of fuzzy differentiability. Later on, Kaleva^36^ presented fuzzy differentiation and integration. Kandel and Byatt^37^ introduced the FDEs in 1987. FDEs on the nth order differential equation with fuzzy initial conditions were applied by Buckley and Feuring^38,39^. FDE was utilized by Abdi and Allahviranloo^40^ to solve the fuzzy Poisson's equation with fuzzy Dirichlet boundary conditions using the fuzzy finite difference method (FFDM). With the use of double parametric FN, Almutairi et al.^41^ derived the numerical solution of the fuzzy wave equation. Salahsour et al.^42^ studied the fuzzy logistic equation and alley effect using FDE with the help of TFNs. Shehu and Zhao^43^ introduced the homotopy analysis Shehu transform method for the fractional and integral order derivatives using FDEs. Biswal et al.^44^ studied the natural convection of third-grade nanofluid flow between the two parallel plates using HPM in a fuzzy environment. The volume fraction of nanoparticles is considered as a triangular fuzzy number and also shows that the fuzzy result is better than a crisp result. Borah et al.^45^ deliberated the magnetic flow of second-grade fluid in a fuzzy environment using fractional derivatives Caputo-Fabrizio and Atangana-Baleanu. The non-dimensional governing equations are converted into fuzzified governing equations with the help of the Zadeh extension principle and TFN. Later on, Barhoi et al.^46^ studied the impact of second-order slip and magnetic flow on the viscous fluid model through a permeable shrinking sheet under the fuzzy environment. Also, various investigators have applied FST to obtain good results in the field of science and engineering. Such as the Fuzzy HIV model^47^, Predator–prey model^48^, Growth model^49^, Population dynamics model^50^, Application of fuzzy Laplace transform^51^, Fuzzy Integro-differential equation^52^, Giving up smoking model^53^, Fuzzy chemostat model^54^, Fuzzy dengue virus model^55^, fuzzy fractional PDEs^56^, Fuzzy epidemic model^57^, Uncertain conjugate heat transfer^58^ and Transient heat transfer problem^59^.

Nanofluids and hybrid nanofluids are very significant in heat transfer applications when compared with traditional fluids. The maximum existing literature is concerned with the distribution of the solid nanomaterials in the Newtonian fluids and very few articles are available about the solid nanomaterial’s distribution in the non-Newtonian fluids. The present work aims to investigate the heat transfer features and flow of a hybrid special second-grade nanofluid through a convectively permeable shrinking/extending sheet for the stable dispersion of the solid nanomaterials ${\text{Al}}_{{2}} {\text{O}}_{{3}} {\text{ + Cu}}$ with Sodium Alginate as a host fluid. The novelty of this work is listed below,

- The nonlinear thermal radiation, viscous dissipation, and heat source/sink are involved in the heat equation.
- The addition of magnetic flux in the flow of the region is very important in controlling the dynamic behavior in the production process.
- The fuzzy differential equations are formed to calculate the epistemic uncertain dispersal volume fraction of nanomaterials. So, the volume fractions of nanoparticles consider as fuzzy numbers or triangular fuzzy numbers with the help of the $\gamma {\text{ - cut}}$ technique, and $\gamma {\text{ - cut}}$ is discussed through the fuzzy triangular membership functions.
- For the comparison between nanofluids and hybrid nanofluids, we used fuzzy triangular membership functions.

## Problem formulations

**Figure 1:**
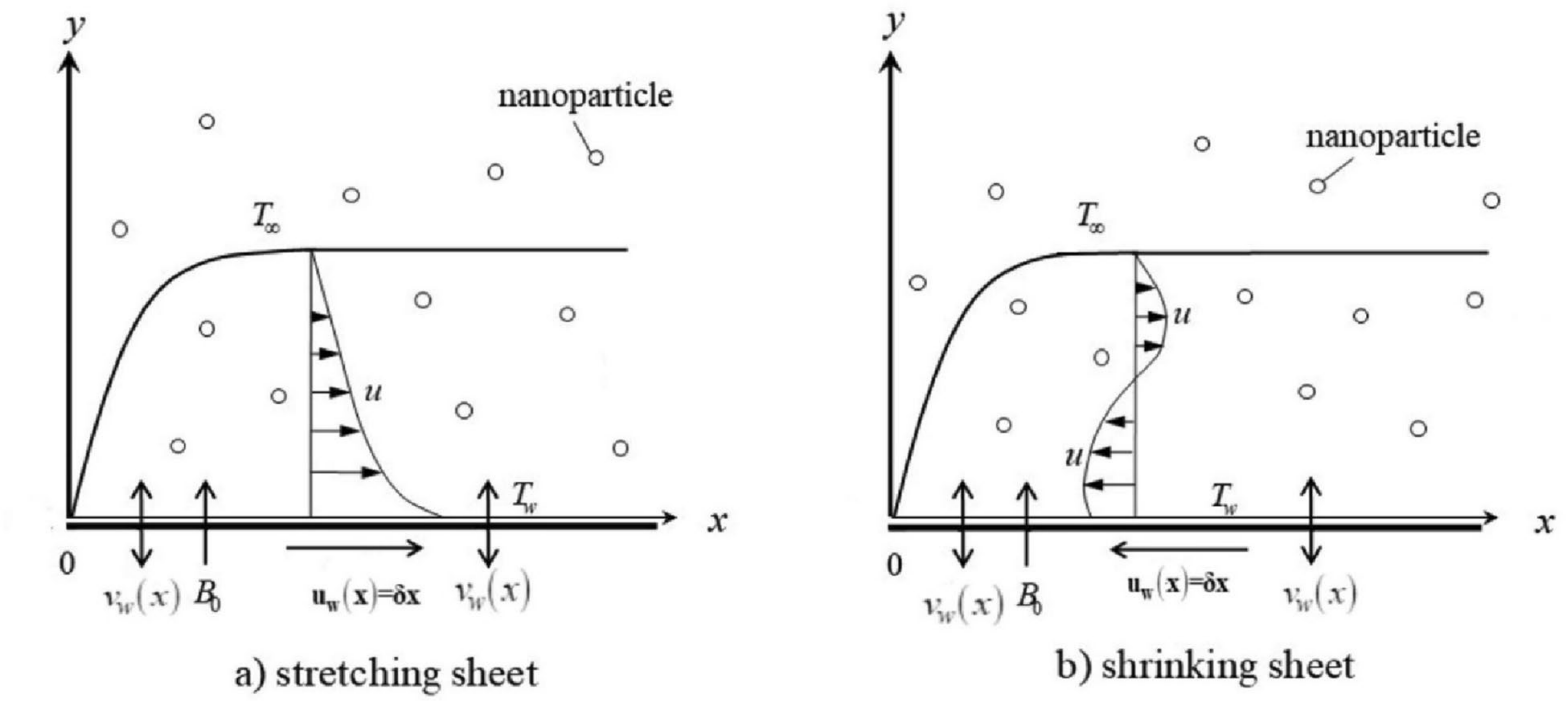
Flow Problem.

An incompressible steady two-dimensional,ilaminar,iboundary-layer and stagnationipointiflow of a non-Newtonian electrically conducting second-gradeihybridinanofluid ${\text{Al}}_{{2}} {\text{O}}_{{3}} {\text{ + Cu/SA}}$ is studied over a convectivelyiheated permeable shrinking/stretchingisheet with nonlinearithermal radiationiand viscousidissipation. In Fig. 1, the flow is examined in the region of y > 0. $v_{w}$ is the mass flux velocity, $B_{0}$ is a constantimagnetic fieldinormal to the sheet, $T_{\infty }$ is the ambient temperature and the temperature of the sheet is $T_{w} .$ Also, assumedithat the sheetimoves with a velocity $u_{w} \left( x \right) = ax$ dependingion $\,a < 0\,\,\,{\text{or}}\,\,a > 0$ for shrinking or stretching sheets respectively. The physicaliproperties of the thermaliconductivity, heat capacity, and viscosity of the host fluid along with nanomaterials vary significantly.

The physical flow problem and governing equations for a specific sort of second-grade hybrid nanofluid were considered by the researchers^18,31^.

1 $$ \frac{\partial v}{{\partial y}} = - \frac{\partial u}{{\partial x}}, $$

2 $$ v\frac{\partial u}{{\partial y}} + u\frac{\partial u}{{\partial x}} = u_{e} \frac{{\partial u_{e} }}{\partial x} + \frac{{2\alpha_{2} }}{{\rho_{hnf} }}\frac{\partial u}{{\partial y}}\frac{{\partial^{2} u}}{\partial x\partial y} + \frac{{\mu_{hnf} }}{{\rho_{hnf} }}\frac{{\partial^{2} u}}{{\partial y^{2} }} - \frac{{\sigma_{hnf} }}{{\rho_{hnf} }}B_{0}^{2} \left( { - u_{e} + u} \right) + g\left( {\beta_{T} } \right)_{hnf} \left( {T - T_{\infty } } \right), $$

3 $$ \begin{gathered} u\frac{\partial T}{{\partial x}} + v\frac{\partial T}{{\partial y}} = \alpha_{hnf} \frac{{\partial^{2} T}}{{\partial y^{2} }} + \frac{{Q_{0} }}{{\left( {\rho c_{P} } \right)_{hnf} }}\left( {T - T_{\infty } } \right) - \frac{1}{{\left( {\rho c_{P} } \right)_{hnf} }}\frac{{\partial q_{r} }}{\partial y} + \frac{{\mu_{hnf} }}{{\left( {\rho c_{P} } \right)_{hnf} }}\left( {\frac{\partial u}{{\partial y}}} \right)^{2} \hfill \\ \,\, \quad \quad \quad \quad \quad \quad + \frac{{\alpha_{2} }}{{\left( {\rho c_{P} } \right)_{hnf} }}\frac{\partial u}{{\partial y}}\left\{ {\frac{\partial }{\partial y}\left( {u\frac{\partial u}{{\partial x}} + v\frac{\partial u}{{\partial y}}} \right)} \right\}, \hfill \\ \end{gathered} $$

where $u_{0} = bx$ with *b* as a constant, is the freeistreamivelocity in outsideiof theiboundary.

The appropriate initial and boundaryiconditions of theiproblem are

4 $$ \begin{gathered} v = v_{w} ,\,\,\,\,\,\,\,u = u_{w} \left( x \right),\,\,\,\,\,\,T = T_{w} \,\,\,\,\,\,\,\,{\text{at}}\,\,\,\,\,\,\,\,y\, = \,0, \hfill \\ u = u_{e} \left( x \right),\,\,\,\,\,\,\,\,\,\,\,\,\,\,\,\,\,\,\,\,\,\,\,\,\,\,T = T_{\infty } \,\,\,\,\,\,\,\,\,\,{\text{as}}\,\,\,\,\,y\, \to \infty .\,\,\, \hfill \\ \end{gathered} $$

Thermo-physical features of the hybrid nanofluids are exposed in the following equation^31^:

5 $$ \alpha_{hnf} = \frac{{k_{hnf} }}{{\left( {\rho c_{P} } \right)_{hnf} }},\mu_{nf} = \mu_{f} \left( {1 - \phi_{1} } \right)^{ - 2.5} \left( {1 - \phi_{2} } \right)^{ - 2.5} , $$

6 $$ \rho_{hnf} = \left[ {\left( {1 - \phi_{2} } \right)\left\{ {\rho_{f} \left( {1 - \phi_{1} } \right) + \rho_{{s_{1} }} \phi_{1} } \right\} + \rho_{{s_{2} }} \phi_{2} } \right], $$

7 $$ \left( {\rho C_{\rho } } \right)_{hnf} = \left[ {\left( {\rho C_{\rho } } \right)_{f} \left( {1 - \phi_{1} } \right) + \left( {\rho C_{\rho } } \right)_{{s_{1} }} \phi_{1} } \right]\left( {1 - \phi_{2} } \right), $$

8 $$ \frac{{k_{hnf} }}{{k_{nf} }} = \frac{{2k_{nf} - 2\phi_{1} \left( {k_{{s_{1} }} - k_{nf} } \right) + k_{{s_{1} }} }}{{2k_{nf} + \phi_{1} \left( {k_{{s_{1} }} - k_{nf} } \right) + k_{{s_{1} }} }},\quad \frac{{k_{nf} }}{{k_{f} }} = \frac{{2k_{f} - 2\phi_{2} \left( {k_{{s_{2} }} - k_{f} } \right) + k_{{s_{2} }} }}{{2k_{f} + \phi_{2} \left( {k_{{s_{2} }} - k_{f} } \right) + k_{{s_{2} }} }}, $$

9 $$ \left( {\beta_{T} } \right)_{hnf} = \phi_{2} \left( {\beta_{T} } \right)_{{s_{2} }} + \left( {1 - \phi_{2} } \right)\left[ {\left( {1 - \phi_{1} } \right)\left( {\beta_{T} } \right)_{f} + \phi_{1} \left( {\beta_{T} } \right)_{{s_{1} }} } \right], $$

10 $$ \sigma_{hnf} = \left[ {\frac{{\sigma_{{s_{2} }} \left( {1 + 2\phi_{2} } \right) + 2\sigma_{bf} \left( {1 - \phi_{2} } \right)}}{{\sigma_{{s_{2} }} \left( {1 - \phi_{2} } \right) + \sigma_{bf} \left( {2 + \phi_{2} } \right)}}} \right]\sigma_{bf} ,\sigma_{bf} = \left[ {\frac{{\sigma_{{s_{1} }} \left( {1 + 2\phi_{1} } \right) + 2\sigma_{f} \left( {1 - \phi_{1} } \right)}}{{\sigma_{{s_{1} }} \left( {1 - \phi_{1} } \right) + \sigma_{f} \left( {2 + \phi_{1} } \right)}}} \right]\sigma_{f} , $$

where ${\text{Al}}_{{2}} {\text{O}}_{{3}}$ and ${\text{Cu}}$ are nanoparticles having the volume fractions $\phi_{1}$ and $\phi_{2}$ respectively. Also $s_{1}$ and $s_{2}$ denote the ${\text{Al}}_{{2}} {\text{O}}_{{3}}$ and ${\text{Cu}}$ solid nanoparticles, respectively. The thermophysical properties of SA, Copper and Aluminum oxide are shown in Table 1.

**Table 1:** Thermo-physical properties of base fluid and nanoparticles^14,31^.

Physical properties	$\rho \left( {{\text{kg/m}}^{3} } \right)$	$\rho c_{p} \left( {\text{J/kgK}} \right)$	$k\left( {\text{W/mK}} \right)$	$\beta_{T} \times 10^{ - 5} \left( {1/{\text{K}}} \right)$	$\sigma \left( {\Omega /{\text{m}}} \right)^{ - 1}$
SA	989	4175	0.6376	99	$2.6 \times 10^{ - 4}$
${\text{Al}}_{{2}} {\text{O}}_{{3}}$	3970	765	40	0.85	$3.69 \times 10^{7}$
${\text{Cu}}$	8933	385	401	1.67	$5.96 \times 10^{7}$

Now, introducing the suitable similarity variables^31^ for non-dimensionalizing the governing equations.

11 $$ \eta = \sqrt {{b \mathord{\left/ {\vphantom {b {\nu_{f} }}} \right. \kern-\nulldelimiterspace} {\nu_{f} }}} y,\,\,\psi = \sqrt {b\nu_{f} } xf\left( \eta \right),\,\,T = T_{\infty } + \left( {T_{w} - T_{\infty } } \right)\theta \left( \eta \right),\,\,u = \frac{\partial \psi }{{\partial y}},v = - \frac{\partial \psi }{{\partial x}}. $$

Using Eq. (11) into Eqs. (1), (2), and (3). Equation (1) is equivalently satisfied, while the remaining got the following form:

12 $$ \frac{{\mu_{r} }}{{\rho_{r} }}f^{\prime\prime\prime} + ff^{\prime\prime} - \left( {f^{\prime}} \right)^{2} + \frac{2K}{{\rho_{r} }}\left( {f^{\prime\prime}} \right)^{2} - \frac{{\sigma_{r} }}{{\rho_{r} }}M\left( {f^{\prime} - 1} \right) + \left( {\beta_{T} } \right)_{r} Gr\theta + 1 = 0, $$

13 $$ \begin{gathered} \alpha_{r} \theta^{\prime\prime} + \Pr f\theta^{\prime} + \frac{{{\text{PrH}} \theta }}{{\left( {\rho c_{P} } \right)_{r} }} + \frac{{Nr\theta^{\prime\prime}}}{{\left( {\rho c_{P} } \right)_{r} }}\left\{ {1 + \theta \left( {\theta_{w} - 1} \right)} \right\}^{3} + \frac{{3Nr\left( {\theta^{\prime}} \right)^{2} }}{{\left( {\rho c_{P} } \right)_{r} }}\left( {\theta_{w} - 1} \right)\left\{ {1 + \theta \left( {\theta_{w} - 1} \right)} \right\}^{2} \hfill \\ + \frac{{f^{\prime\prime}{\text{PrEc}} }}{{\left( {\rho c_{P} } \right)_{r} }}\left[ {K\left( {f^{\prime}f^{\prime\prime} - ff^{\prime\prime\prime}} \right) + \mu_{r} f^{\prime\prime}} \right] = 0, \hfill \\ \end{gathered} $$

along with the boundary conditions

14 $$ \begin{gathered} \,f = s,\,\,\,\,f^{\prime} = \beta ,\,\,\theta = 1\,\,\,\,\,{\text{at}}\,  \,\,\eta \, = \,0, \hfill \\ \,\,f^{\prime} = 1,\,\,\theta = 0\,\,\,\,  {\text{as}}\,  \,\,\eta \, \to \infty ,\,\,\, \hfill \\ \end{gathered} $$

where, $\sigma_{r} = {{\sigma_{hnf} } \mathord{\left/ {\vphantom {{\sigma_{hnf} } {\sigma_{f} ,}}} \right. \kern-\nulldelimiterspace} {\sigma_{f} ,}}$ $\mu_{r} = {{\mu_{hnf} } \mathord{\left/ {\vphantom {{\mu_{hnf} } {\mu_{f} ,}}} \right. \kern-\nulldelimiterspace} {\mu_{f} ,}}$ $\left( {\beta_{T} } \right)_{r} = {{\left( {\beta_{T} } \right)_{hnf} } \mathord{\left/ {\vphantom {{\left( {\beta_{T} } \right)_{hnf} } {\left( {\beta_{T} } \right)_{f} ,}}} \right. \kern-\nulldelimiterspace} {\left( {\beta_{T} } \right)_{f} ,}}$ $\rho_{r} = {{\rho_{hnf} } \mathord{\left/ {\vphantom {{\rho_{hnf} } {\rho_{f} ,}}} \right. \kern-\nulldelimiterspace} {\rho_{f} ,}}$ $\alpha_{hnf} = {{k_{hnf} } \mathord{\left/ {\vphantom {{k_{hnf} } {\left( {\rho c_{P} } \right)_{hnf} ,}}} \right. \kern-\nulldelimiterspace} {\left( {\rho c_{P} } \right)_{hnf} ,}}$ $\alpha_{r} = {{\alpha_{hnf} } \mathord{\left/ {\vphantom {{\alpha_{hnf} } {\alpha_{f} ,\,}}} \right. \kern-\nulldelimiterspace} {\alpha_{f} ,\,}}$ $\left( {\rho c_{P} } \right)_{r} = {{\left( {\rho c_{P} } \right)_{hnf} } \mathord{\left/ {\vphantom {{\left( {\rho c_{P} } \right)_{hnf} } {\left( {\rho c_{P} } \right)_{f} ,\,}}} \right. \kern-\nulldelimiterspace} {\left( {\rho c_{P} } \right)_{f} ,\,}}$ $Gr = {{\left( {T_{w} - T_{\infty } } \right)g\left( {\beta_{T} } \right)_{f} } \mathord{\left/ {\vphantom {{\left( {T_{w} - T_{\infty } } \right)g\left( {\beta_{T} } \right)_{f} } {bx^{2} }}} \right. \kern-\nulldelimiterspace} {bx^{2} }},$ $M = {{\sigma_{f} B_{0}^{2} } \mathord{\left/ {\vphantom {{\sigma_{f} B_{0}^{2} } {b\rho_{f} }}} \right. \kern-\nulldelimiterspace} {b\rho_{f} }},$ ${{K = b\alpha_{2} } \mathord{\left/ {\vphantom {{K = b\alpha_{2} } {\mu_{f} }}} \right. \kern-\nulldelimiterspace} {\mu_{f} }},$ $\Pr = {{v_{f} } \mathord{\left/ {\vphantom {{v_{f} } {\alpha_{f} }}} \right. \kern-\nulldelimiterspace} {\alpha_{f} }},$ $H = {{Q_{0} } \mathord{\left/ {\vphantom {{Q_{0} } {b\left( {\rho c_{P} } \right)_{f} }}} \right. \kern-\nulldelimiterspace} {b\left( {\rho c_{P} } \right)_{f} }},$ $\theta_{w} = {{T_{w} } \mathord{\left/ {\vphantom {{T_{w} } {T_{\infty } }}} \right. \kern-\nulldelimiterspace} {T_{\infty } }},$ $Nr = {{16\sigma^{*} T_{\infty }^{3} } \mathord{\left/ {\vphantom {{16\sigma^{*} T_{\infty }^{3} } {3k_{f} k^{*} v_{f} }}} \right. \kern-\nulldelimiterspace} {3k_{f} k^{*} v_{f} }},$ $Ec = {{\left( {bx} \right)^{2} } \mathord{\left/ {\vphantom {{\left( {bx} \right)^{2} } {\left( {\rho c_{p} } \right)_{f} \left( {T_{w} - T_{\infty } } \right)}}} \right. \kern-\nulldelimiterspace} {\left( {\rho c_{p} } \right)_{f} \left( {T_{w} - T_{\infty } } \right)}},$ $\beta = {a \mathord{\left/ {\vphantom {a b}} \right. \kern-\nulldelimiterspace} b}$ the velocity ratio parameter, $v_{w} \left( x \right) = - s\sqrt {bv_{f} }$ and *s* is the rate of mass transfer through the permeable sheet.

The skin-friction coefficient $Cf_{x}$ and the Nusselt number $Nu_{x}$ are two important physical quantities of interest defined as

15 $$ Cf_{x} = \frac{1}{{\rho_{f} u_{e}^{2} }}\left[ {\mu_{hnf} \frac{\partial u}{{\partial y}} + \alpha_{2} \left\{ {2\frac{\partial u}{{\partial y}}\frac{\partial u}{{\partial x}} + u\frac{{\partial^{2} u}}{\partial x\partial y}} \right\}} \right]_{y = 0} . $$

16 $$ Nu_{x} = \frac{x}{{k_{f} \left( {T_{w} - T_{\infty } } \right)}}\left[ { - k_{hnf} \frac{\partial T}{{\partial y}} - \frac{{16\sigma^{*} T_{\infty }^{3} }}{{3k^{*} }}\frac{\partial T}{{\partial y}}} \right]_{y = 0} . $$

Using Eq. (11) into Eqs. (15) and (16), we have

17 $$ {\text{Re}}_{x}^{{{\raise0.7ex\hbox{$1$} \!\mathord{\left/ {\vphantom {1 2}}\right.\kern-\nulldelimiterspace} \!\lower0.7ex\hbox{$2$}}}} Cf_{x} = \left( {\mu_{r} + Kf^{\prime}\left( 0 \right)} \right)f^{\prime\prime}\left( 0 \right)\;{\text{and}}\quad {\text{Re}}_{x}^{{ - {\raise0.7ex\hbox{$1$} \!\mathord{\left/ {\vphantom {1 2}}\right.\kern-\nulldelimiterspace} \!\lower0.7ex\hbox{$2$}}}} Nu_{x} = - \left( {k_{r} + Nr} \right)\theta^{\prime}\left( 0 \right), $$

where ${\text{Re}}_{x} = {{xu_{w} } \mathord{\left/ {\vphantom {{xu_{w} } {v_{f} }}} \right. \kern-\nulldelimiterspace} {v_{f} }}$ is the local Reynolds number.

## Fuzzy analysis

### Definition 2.1

^32^: “A fuzzy set is defined as the set of ordered pairs such that $\overline{\Omega } = \left\{ {\left( {\eta ,\mu_{{\overline{\Omega }}} (\eta )} \right):\eta \in X,\mu_{{\overline{\Omega }}} (\eta ) \in \left[ {0,\,1} \right]} \right\},$ where $X$ is the universal set, $\mu_{{\overline{\Omega }}} (\eta )$ is the MF or membership level of $\overline{\Omega }$ and mapping defined as $\mu_{{\overline{\Omega }}} (\eta ):X \to [0,\,1].$ Also, the values of $\mu_{{\overline{\Omega }}} (\eta )$ varies from 0 to 1. If $\mu_{{\overline{\Omega }}} (\eta ) = 0$ means that $\eta$ does not belong to the fuzzy set, however, $\mu_{{\overline{\Omega }}} (\eta ) = 1$ implies that $\eta$ relates to the fuzzy set and if $0 < \mu_{{\overline{\Omega }}} (\eta ) < 1$ which means that the membership level of $\eta$ is uncertain.”

### Definition 2.2

^32,35^: The $\gamma {\text{ - cut}}$ or $\gamma {\text{ - level}}$ of a fuzzy set $\overline{\Omega },$ is a crisp set $U_{\gamma }$ and defined by $U_{\gamma } = \left\{ {\eta /\mu_{{\overline{\Omega }}} (\eta ) \ge \gamma } \right\},$ where $0 \le \gamma \le 1.$

### Definition 2.3

^33^: The fuzzy set $\overline{\Omega }$ defined on the universal set of real number R, is said to be a FN, which satisfies the following properties:

(i)$\mu_{{\overline{\Omega }}} \left( \eta \right)$ is piecewise continuous. (ii) $\overline{\Omega }$ is convex. (iii) $\overline{\Omega }$ is normal i.e., $\exists \,\,y_{0} \in R$ such that $\mu_{{\overline{\Omega }}} (\eta ) = 1.$ (iv) Support of $\overline{\Omega }$ must be bounded.

### Definition 2.4

^34,35^: Let $\overline{\Omega } = (\chi_{1} ,\chi_{2} ,\chi_{3} )$ with MF $\mu_{{\overline{\Omega }}} (\eta )$ is called a MF of TFN if

$$ \mu_{{\overline{\Omega }}} (\eta ) = \left\{ \begin{gathered} \frac{{\eta - \chi_{1} }}{{\chi_{2} - \chi_{1} }}\,\,\,\,\,{\text{for}}\,\,\,\,\eta \in [\chi_{1} ,\,\,\chi_{2} ], \hfill \\ \,\frac{{\eta - \chi_{3} }}{{\chi_{2} - \chi_{3} }}\,\,\,\,{\text{for}}\,\,\,\,\eta \in [\chi_{2} ,\,\,\chi_{3} ], \hfill \\ \,\,\,\,\,\,0,\,\,\,\,\,\,\,\,\,\,\,\,\,\,\,\,{\text{otherwise}}{.} \hfill \\ \end{gathered} \right. $$

**Figure 2:**
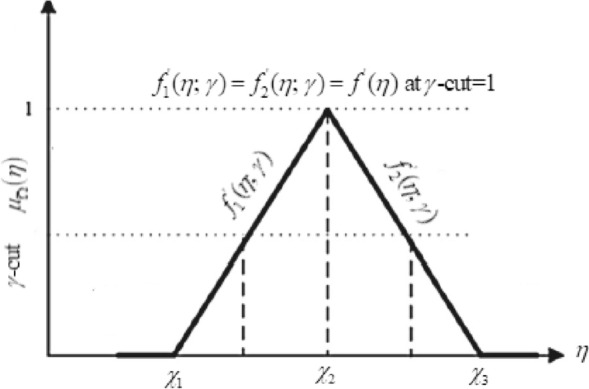
Membership functions of a TFN.

The MF is the building block of FST and fuzziness in an FST is defined by its MF. They have different shapes such that triangular, Gaussian, and trapezoidal. The *x*-axis indicates the universe of discourse, while the *y*-axis shows the degrees of membership in the [0,1] interval. The MF of TFN with peak (or center) $\chi_{2} ,$ left width $\chi_{2} - \chi_{1} > 0,$ right width $\chi_{3} - \chi_{2} > 0$ and these TFNs are transformed into interval numbers through $\gamma {\text{ - cut}}$ approach, is written as $\overline{\Omega } = \left[ {f_{1}^{^{\prime}} (\eta ;\,\gamma ),\,\,f_{2}^{^{\prime}} (\eta ;\,\gamma )} \right] = \left[ {\chi_{1} + (\chi_{2} - \chi_{1} )\gamma ,\,\,\chi_{3} - (\chi_{3} - \chi_{2} )\gamma } \right],$ where $0 \le \gamma \le 1.$ A TFN $\overline{\Omega } = (\chi_{1} ,\chi_{2} ,\chi_{3} )$ and $\gamma {\text{ - cut}}$ of MF see in Fig. 2. An arbitrary TFN satisfies the following conditions:

(i) $f_{1}^{^{\prime}} (\eta ;\,\,\gamma )$ is an increasing function on [0,1]. (ii) $f_{2}^{^{\prime}} (\eta ;\,\,\gamma )$ is a decreasing function on [0,1]. (iii) $f_{1}^{^{\prime}} (\eta ;\,\,\gamma ) \le f_{2}^{^{\prime}} (\eta ;\,\,\gamma )$ on [0, 1]. (iv) $f_{1}^{^{\prime}} (\eta ;\,\,\gamma )$ and $f_{2}^{^{\prime}} (\eta ;\,\,\gamma )$ are bounded on left continuous and right continuous at [0, 1] respectively. (v) If $f_{1}^{^{\prime}} (\eta ;\,\,\gamma ) = f_{2}^{^{\prime}} (\eta ;\,\,\gamma ) = f^{^{\prime}} (\eta )$ where $f^{^{\prime}} (\eta ,\,\,1)$ becomes crisp at $\gamma {\text{ - cut}} = 1$ and frequently used in the fuzzy physical analysis.

$U_{\gamma }$ must be closed interval for every $0 \le \gamma \le 1$ also $\gamma$, is called level of credibility or presumption. MF or grade is also named as the grade of possibility or grade of credibility for a given number. So, the triangular fuzzy uncertainty is defined as $f_{1}^{^{\prime}} (\eta ;\,\gamma )$ (lower bound), $f^{^{\prime}} (\eta ,\,\,1)$ (most belief value) and $f_{2}^{^{\prime}} (\eta ;\,\gamma )$ (upper bound).

### Definition 2.5

^35–40^: Let I be a real interval. A mapping $\tilde{u}:I \to F$ is called a fuzzy process, defined as $\overline{f}^{^{\prime}} (\eta ;\,\gamma ) = \left[ {f_{1}^{^{\prime}} (\eta ;\,\gamma ),f_{2}^{^{\prime}} (\eta ;\,\gamma )} \right],\,\,\eta \in I\,$ and $\gamma \in [0,\,1].$ The derivative $\frac{{d\overline{f}^{^{\prime}} (\eta ;\,\gamma )}}{d\eta } \in F$ of a fuzzy process $\overline{f}^{^{\prime}} (\eta ;\,\gamma )$ is defined by $\frac{{d\overline{f}^{^{\prime}} (\eta ;\,\gamma )}}{d\eta } = \left[ {\frac{{df_{1}^{^{\prime}} (\eta ;\,\gamma )}}{d\eta },\,\frac{{df_{2}^{^{\prime}} (\eta ;\,\gamma )}}{d\eta }} \right].$

### Definition 2.6

^35–40^: Let $I \subseteq R,\,\,\overline{f}^{^{\prime}} \left( {\eta ,\,\gamma } \right)$ be a fuzzy valued function define on I. Let $\overline{f}^{^{\prime}} (\eta ;\,\gamma ) = \left[ {f_{1}^{^{\prime}} (\eta ;\,\gamma ),f_{2}^{^{\prime}} (\eta ;\,\gamma )} \right]\,$ for all $\gamma$-cut. Assume that $f_{1}^{^{\prime}} (\eta ;\,\gamma )$ and $f_{2}^{^{\prime}} (\eta ;\,\gamma )$ have continuous derivatives or differentiable, for all $\eta \in I$ and $\gamma$ then $\left[ {\frac{{d\overline{f}^{^{\prime}} (\eta ;\,\gamma )}}{d\eta }} \right]_{\gamma } = \left[ {\frac{{df_{1}^{^{\prime}} (\eta ;\,\gamma )}}{d\eta },\frac{{df_{2}^{^{\prime}} (\eta ;\,\gamma )}}{d\eta }} \right]_{\gamma } .$ Similarly, we can define higher-order ordinary derivatives in the same way. A FN by an ordered pair of functions $\left[ {\frac{{d\overline{f}^{^{\prime}} (\eta ;\,\gamma )}}{d\eta }} \right]_{\gamma } ,$ satisfy the following conditions:

(i) $\frac{{df_{1}^{^{\prime}} (\eta ;\,\gamma )}}{d\eta }$ and $\frac{{df_{2}^{^{\prime}} (\eta ;\,\gamma )}}{d\eta }$ are continuous on [0, 1]. (ii) $\frac{{df_{1}^{^{\prime}} (\eta ;\,\gamma )}}{d\eta }$ is an increasing function on [0, 1]. (iii)$\frac{{df_{2}^{^{\prime}} (\eta ;\,\gamma )}}{d\eta }$ is a decreasing function on [0, 1]. (iv) $\frac{{df_{1}^{^{\prime}} (\eta ;\,\gamma )}}{d\eta } \le \frac{{df_{2}^{^{\prime}} (\eta ;\,\gamma )}}{d\eta }$ on [0, 1]. (v) If $\frac{{df_{1}^{^{\prime}} (\eta ;\,\,\gamma )}}{d\eta } = \frac{{df_{2}^{^{\prime}} (\eta ;\,\,\gamma )}}{d\eta } = \frac{{df^{^{\prime}} (\eta )}}{d\eta },$ where $f^{^{\prime}} (\eta ,\,\,1)$ becomes a crisp at $\gamma {\text{ - cut}} = 1$.

## Formulation of the crisp problem into the fuzzy problem using FDEs

First, we will go over some fuzzy basics before comparing nanofluid and hybrid nanofluid. The velocity and temperature are affected by the small change in the value of the volume fraction of nanoparticles. Some researchers take the volume fraction of nanoparticles in the range of [0.01–0.04], so the flow rate and heat transfer of the nanofluid just depends on these values. Thus, uncertainty arises due to the fixed crisp values of the volume fractions of nanoparticles. Since $\phi_{1}$ and $\phi_{2}$ represents the volume fraction of ${\text{Al}}_{{2}} {\text{O}}_{{3}}$ and ${\text{Cu}}$ respectively, so it is better to handle a complicated problem in a fuzzy environment by taking both volume fractions as FN. In this investigation, the volume fractions of nanoparticles are considered as FNs or TFNs and the TFNs are converted into $\gamma {\text{ - cut}}$ techniques as exposed in Table 2**.**

**Table 2:** TFNs of fuzzy nanoparticles of volume fraction.

Fuzzy numbers	Crisp value	TFN	$\gamma {\text{ - cut}}$ approach
$\phi_{1}$ $\left( {{\text{Al}}_{{2}} {\text{O}}_{{3}} } \right)$	[0.01–0.04]	[0, 0.1, 0.2]	$\left[ {0.1\gamma ,\,\,0.2 - 0.1\gamma } \right],\,\gamma \in \left[ {0,\,1} \right]$
$\phi_{2}$ $\left( {{\text{Cu}}} \right)$	[0.01–0.04]	[0, 0.1, 0.2]	$\left[ {0.1\gamma ,\,\,0.2 - 0.1\gamma } \right],\,\gamma \in \left[ {0,\,1} \right]$

The TFN defines the variation of FN at each $\gamma {\text{ - cut}}{.}$ The TFNs are used to describe the triangular MFs of the FNs which is ranging from 0 to 1 see Fig. 2. These specified ranges are generally used to build up the current problem. The FNs contain left monotonically non-decreasing and right monotonically non-increasing functions respectively, which make the triangular shape. Our purpose is to establish a comparison of nanofluid and hybrid nanofluid through the triangular MF.

For the fuzzy solution, the governing coupled non-linear differential Eqs. (12)–(14) can be converted into FDEs using $\gamma {\text{ - cut}}$ the approach that controls the fuzzy solution. So according to the Definition 2.5 and Definition 2.6 we have

18 $$ \begin{gathered} \frac{{\mu_{r} }}{{\rho_{r} }}\frac{{d^{3} f\left( {\eta ,\,\gamma } \right)}}{{d\eta^{3} }} + f\left( {\eta ,\,\gamma } \right)\frac{{d^{2} f\left( {\eta ,\,\gamma } \right)}}{{d\eta^{2} }} + \frac{2K}{{\overline{\rho }_{r} }}\left( {\frac{{d^{2} f\left( {\eta ,\,\gamma } \right)}}{{d\eta^{2} }}} \right)^{2} - \left( {\frac{{df\left( {\eta ,\,\gamma } \right)}}{d\eta }} \right)^{2} - \frac{{\sigma_{r} }}{{\rho_{r} }}M\left( {\frac{{df\left( {\eta ,\,\gamma } \right)}}{d\eta } - 1} \right) \hfill \\ + 1 + \left( {\beta_{T} } \right)_{r} Gr\theta \left( {\eta ,\,\gamma } \right) = 0,  \,\,\,\,\,\,\,\,\,\,\, \hfill \\ \end{gathered} $$

19 $$ \begin{gathered} \alpha_{r} \theta^{\prime\prime}\left( {\eta ,\,\gamma } \right) + \Pr f\left( {\eta ,\,\gamma } \right)\theta^{\prime}\left( {\eta ,\,\gamma } \right) + \frac{{{\text{PrH}} \theta \left( {\eta ,\,\gamma } \right)}}{{\left( {\rho c_{P} } \right)_{r} }} + \frac{{Nr\theta^{\prime\prime}\left( {\eta ,\,\gamma } \right)}}{{\left( {\rho c_{P} } \right)_{r} }}\left\{ {1 + \left( {\theta_{w} - 1} \right)\theta \left( {\eta ,\,\gamma } \right)} \right\}^{3} \hfill \\ + \frac{{3Nr\left( {\theta^{\prime}\left( {\eta ,\,\gamma } \right)} \right)^{2} }}{{\left( {\rho c_{P} } \right)_{r} }}\left( {\theta_{w} - 1} \right)\left\{ {1 + \left( {\theta_{w} - 1} \right)\theta \left( {\eta ,\,\gamma } \right)} \right\}^{2} + \frac{{f^{\prime\prime}\left( {\eta ,\,\gamma } \right){\text{PrEc}} }}{{\left( {\rho c_{P} } \right)_{r} }} \hfill \\ \times \left[ {\mu_{r} f^{\prime\prime}\left( {\eta ,\,\gamma } \right) + K\left( {f^{\prime}\left( {\eta ,\,\gamma } \right)f^{\prime\prime}\left( {\eta ,\,\gamma } \right) - f^{\prime\prime\prime}\left( {\eta ,\,\gamma } \right)f\left( {\eta ,\,\gamma } \right)} \right)} \right] = 0, \hfill \\ \end{gathered} $$

along with the boundary conditions

20 $$ \begin{gathered} f^{\prime}\left( {\eta ,\,\gamma } \right) = \beta ,\,\,f\left( {\eta ,\,\gamma } \right) = s,\,\,\theta \left( {\eta ,\,\gamma } \right) = 1,\,\,\,\,\,{\text{at}}\,\,\,\eta \, = \,0, \hfill \\ f^{\prime}\left( {\eta ,\,\gamma } \right) = 1,\,\,\,\theta \left( {\eta ,\,\gamma } \right) = 0,\,\,\,\,\,\,\,\,\,\,\,\,\,\,\,\,\,\,\,\,\,\,\,\,\,\,\,\,\,\,\,\,{\text{as}}\,\,\,\eta \, \to \infty .\,\,\, \hfill \\ \end{gathered} $$

According to the definition of FDEs, the fuzzy velocity profile can be written as $$f^{\prime}(\eta ,\,\gamma )$$$$= \left[ {f^{\prime}_{1} (\eta ,\,\gamma ),\,f^{\prime}_{2} (\eta ,\,\gamma )} \right],\,0 \le \gamma \le 1.$$ Here, $$f^{\prime}_{1} (\eta ,\,\gamma )$$ is lower bound and $$f^{\prime}_{2} (\eta ,\,\gamma )$$ is an upper bound of fuzzy velocity profiles. Similarly, the fuzzy temperature profiles are $$\overline{\theta }(\eta ,\,\gamma ) = \left[ {\theta_{1} (\eta ,\,\gamma ),\,\theta_{2} (\eta ,\,\gamma )} \right],\,0 \le \gamma \le 1.$$

## Results and discussion

In this portion, the significant features of the flow and heat transfer are achieved using second-grade hybrid nanofluids (${\text{Al}}_{{2}} {\text{O}}_{{3}} {\text{ + Cu/SA}}$) passing over a permeable shrinking/stretching sheet. The numerical solutions of non-dimensional governing coupled highly non-linear differential equations are obtained via a built-in numerical technique bvp4c. The numerical results are examined through figures and in the tabular forms for the various values of control dimensionless parameters such as buoyancy ratio parameter (*Gr*), second-grade fluid parameter (*K*), heat source or sink parameter (*H*), magnetic parameter (*M*), rate of mass transfer parameter (*s*), thermal radiation parameter (*Nr*), Prandtl number (*Pr*), temperature ratio parameter $\left( {\theta_{w} } \right),$ Eckert number (*Ec*), velocity ratio parameter $\left( \beta \right),$ and volume fraction of hybrid nanoparticles $\left( {\phi_{1} ,\,\,\phi_{2} } \right)$. The skin-friction coefficient $C_{fx}$ and the Nusselt number $Nu_{x}$ are also calculated and discussed. Table 3 shows a comparison of the results obtained by the current method with those got by Naganthran et al.^16^ Roy and Pop^30^ and Bhattacharyya^7^. The table shows that the current solutions give a good agreement.

**Table 3:** Comparison of $$f^{\prime\prime}\left( 0 \right)$$ for various values of $$K = 0,\,Gr = 0,\,H = 0,\,Nr = 0,\,$$$$Ec = 0,\,M = 0$$ $$\,\,{\text{and}}\,\,s = 0.$$

$\beta$	Naganthran et al.^16^	Bhattacharyya^7^	Roy and Pop^31^	Present
−1.15	1.0822311	1.0822316	1.0822083	1.0822081
−1.20	0.9324733	0.9324728	0.9324443	0.9324442
−1.2465	0.5842759	0.5842915	0.5836674	0.5836661
−1.24657	0.5745397	0.5745268	0.5727295	0.5727290

**Figure 3:**
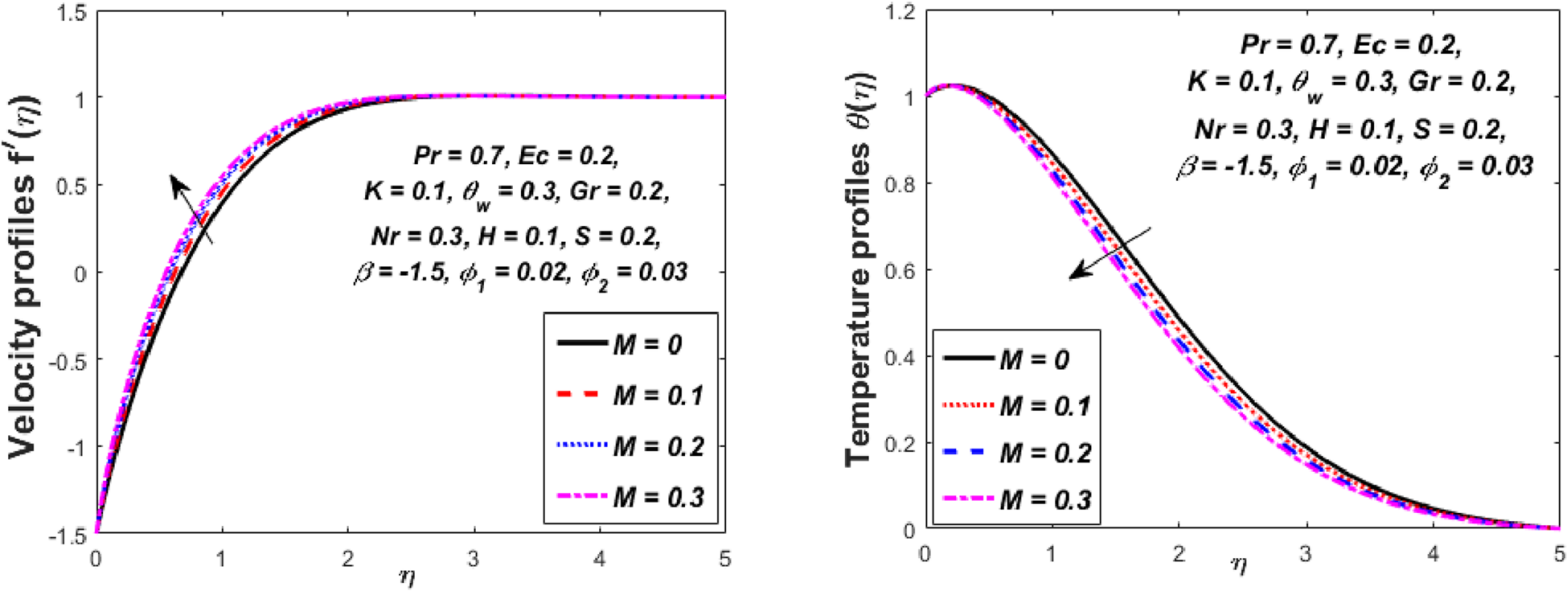
Influence of *M* on velocity $f^{\prime}\left( \eta \right)$ and temperature $\theta \left( \eta \right)$ profiles.

**Figure 4:**
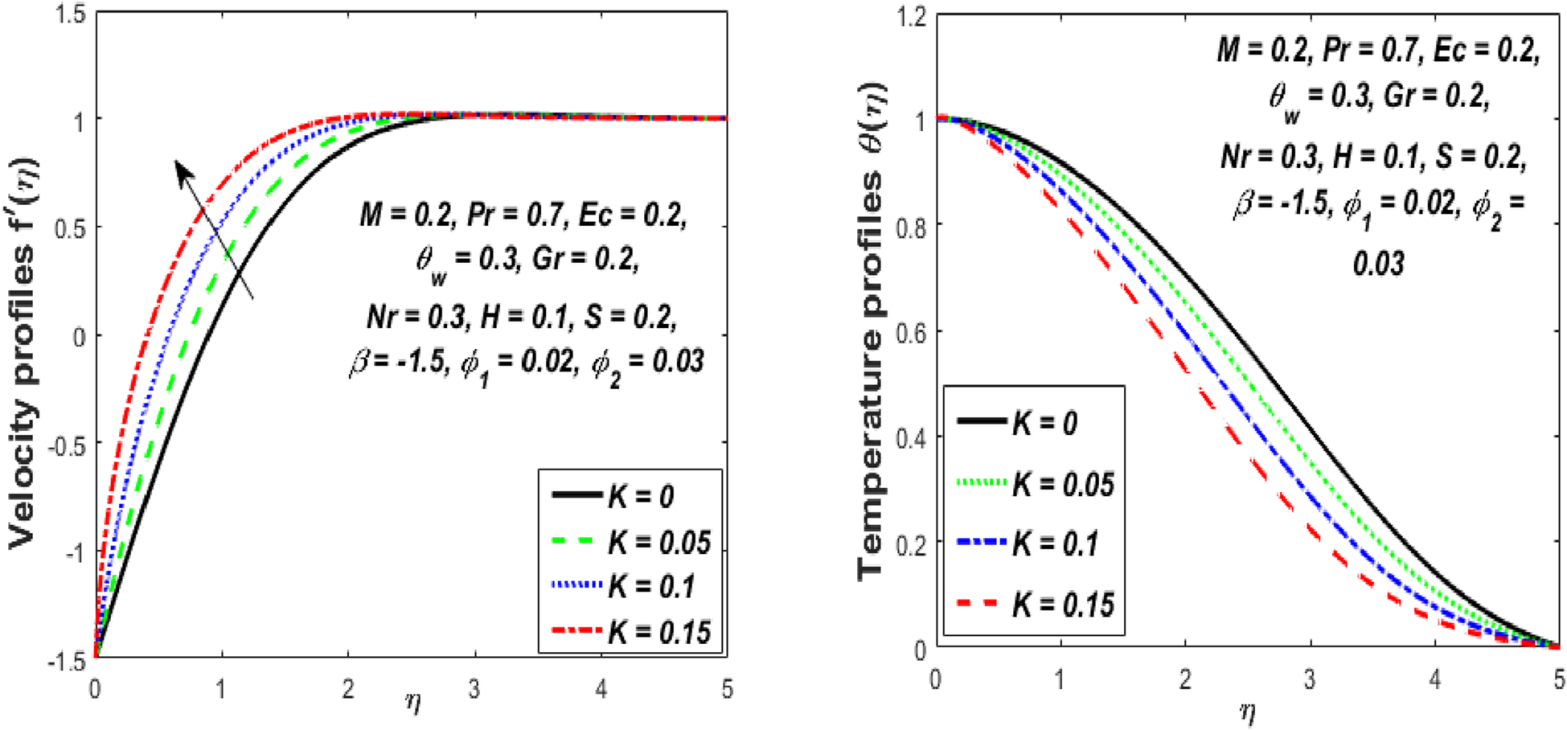
Influence of *K* on velocity $f^{\prime}\left( \eta \right)$ and temperature $\theta \left( \eta \right)$ profiles.

**Figure 5:**
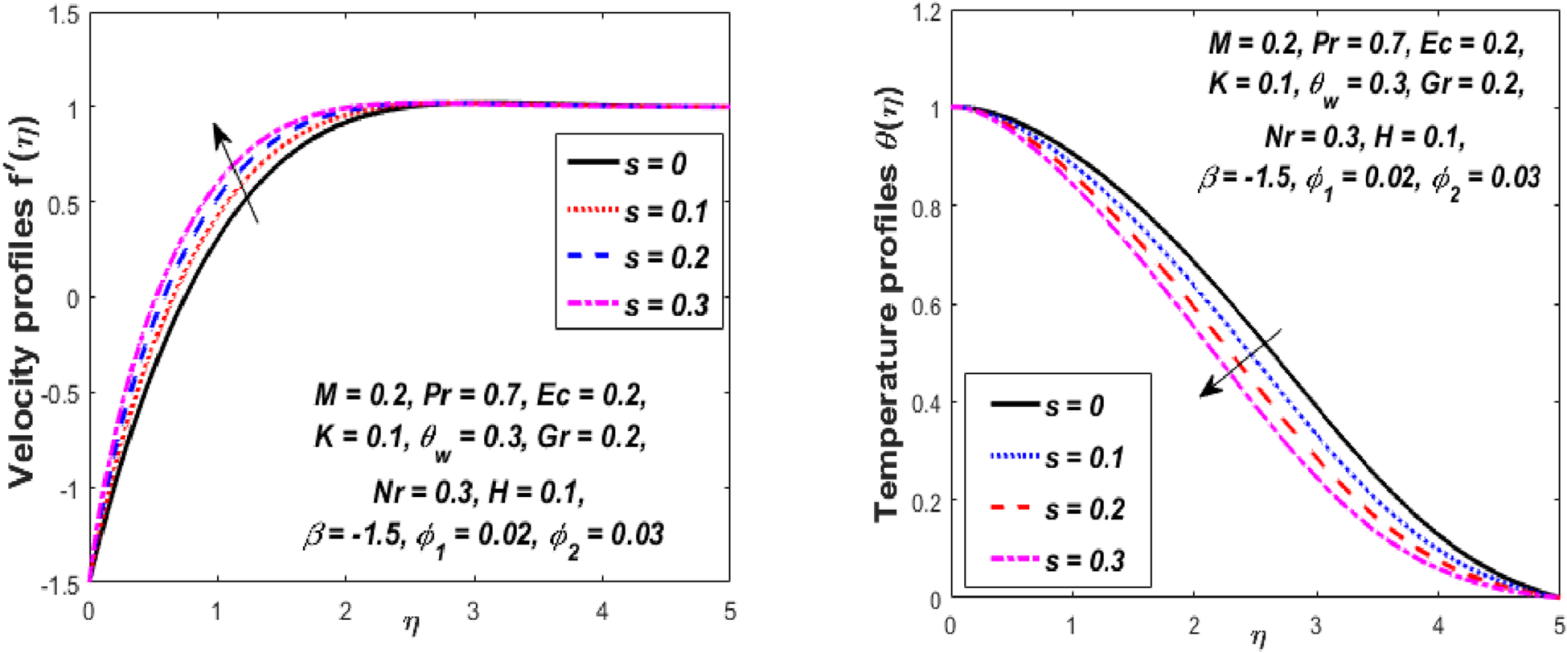
Influence of *s* on velocity $f^{\prime}\left( \eta \right)$ and temperature $\theta \left( \eta \right)$ profiles.

**Figure 6:**
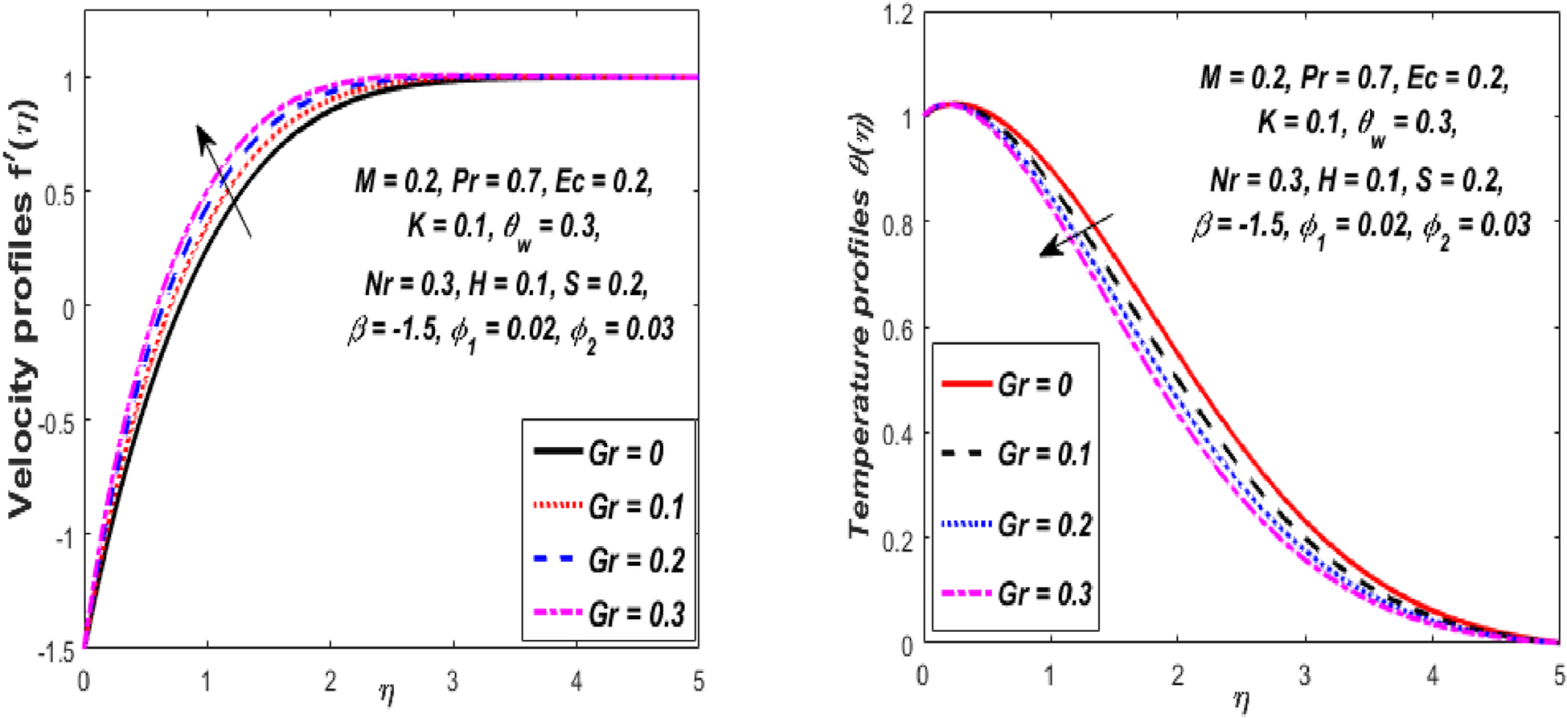
Influence of *Gr* on velocity $f^{\prime}\left( \eta \right)$ and temperature $\theta \left( \eta \right)$ profiles.

**Figure 7:**
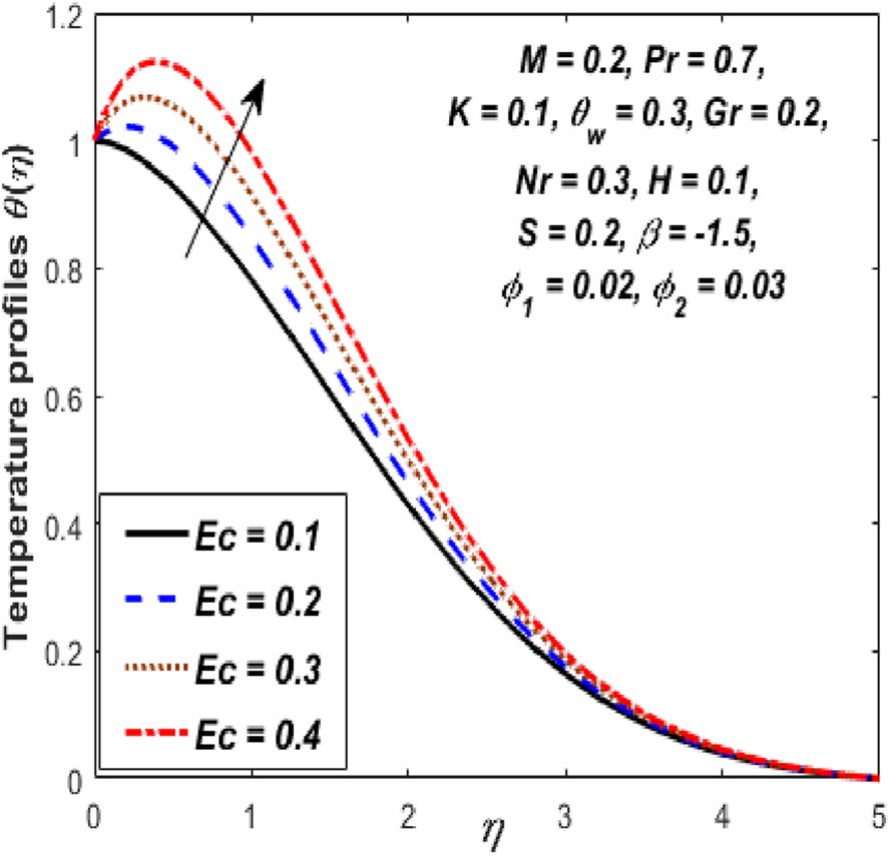
Influence of *Ec* on temperature $\theta \left( \eta \right)$ profile.

**Figure 8:**
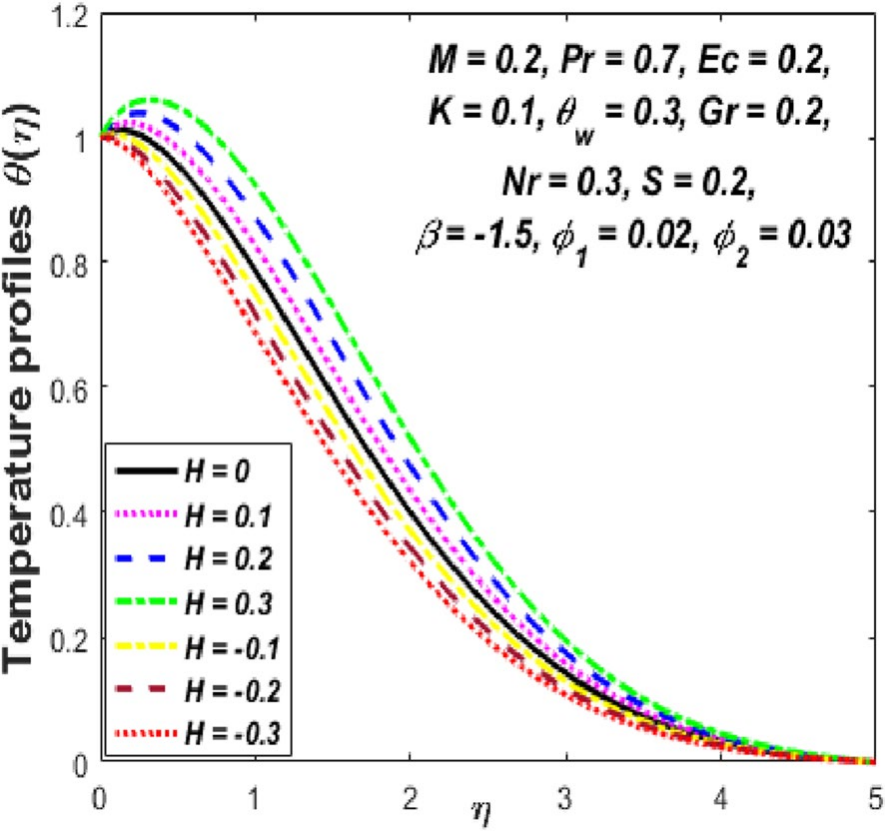
Influence of *H* on temperature $\theta \left( \eta \right)$ profile.

**Figure 9:**
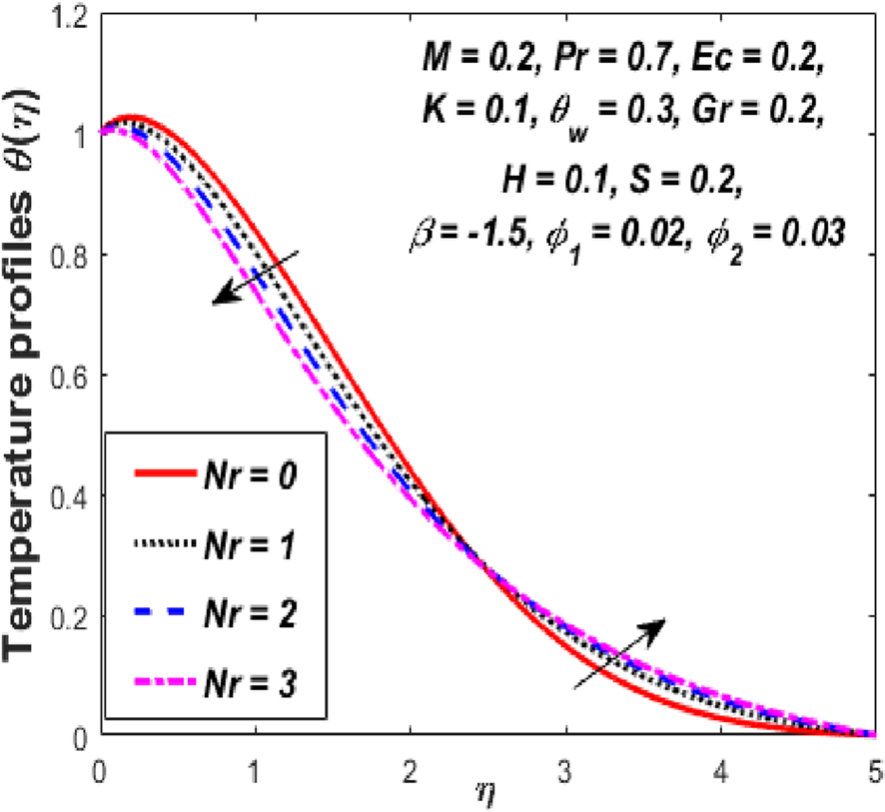
Influence of *Nr* on temperature $\theta \left( \eta \right)$ profile.

**Figure 10:**
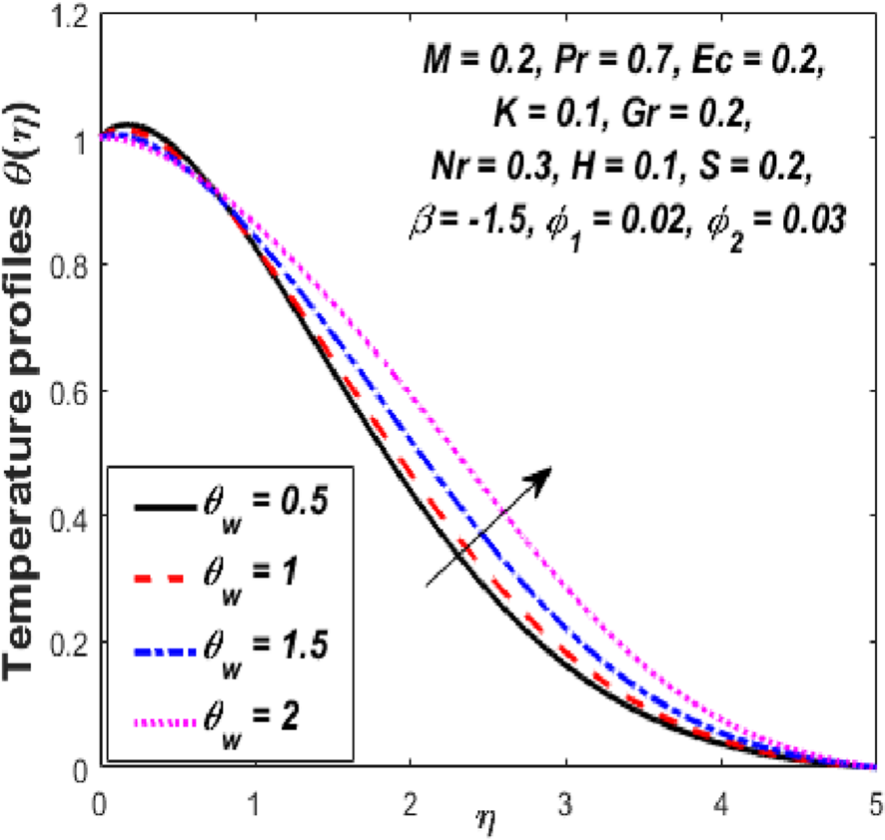
Influence of $\theta_{w}$ on temperature $\theta \left( \eta \right)$ profile.

Figure 3 reports the influence of magnetic parameter (*M)* on the velocity and temperature fields of the hybrid nanofluid for the shrinking sheet case. As the values of *M* increases, the temperature of the nanofluid decreases and the velocity upsurges. Physically, the momentum boundary layer becomes thinner whereas the thermal boundary layer becomes denser because of the increase in the magnetic field intensity. It's because a higher applied magnetic field causes flow velocity to increase by reducing collisions between nanoparticles. Further, the heat energy is discharged into the effective hybrid nanofluid due to the presence of viscous dissipation and heat source impacts. The influences of the second-grade fluid parameter (*K*) on velocity and temperature fields are portrayed in Fig. 4. As the value of K is increased, a notable increase in the velocity field while a decrease in the temperature field in the flow region is observed. The reason is that a considerable decrease in boundary-layer thicknesses due to the larger normal stress expends the force to the adjacent particles because they are enforced to move quickly. Variations of hybrid nanofluid velocity and temperature fields for various values of the suction parameter (s) in the boundary-layer are presented in Fig. 5. The velocity of the hybrid nanofluid rises while the temperature profile of the hybrid nanofluid falls when *s* is elevated. The suction of the hybrid nanofluid creates a vacuum, which necessitates raising the velocity of the hybrid nanofluid. The heat emitted from the sheet owing to fluid movement flows faster to lower the temperature. The influence of the buoyancy force parameter (*Gr*) on the velocity and temperature profiles is depicted in Fig. 6. When *Gr* is boosted, the velocity field in the flow zone increases while the temperature field in the flow area declines. The large values of *Gr* enable the hybrid nanofluid to move faster in the boundary layer. The thermodynamic changes observed in the temperature field for the different values of Eckert number (viscous dissipation effect) (*Ec)* is shown in Fig. 7. The thermal profile is enhanced for the several values of *Ec* in the flow section. Physically, the presence of frictional heating forces (viscous dissipation effect) in the hybrid nanofluid is converted to heat energy and therefore, the temperature profile increases in the boundary-layer region of the shrinking sheet. The combined impact of the heat sink $(H < 0)$ or source $(H > 0)$ parameter on the temperature field is exhibited in Fig. 8. When the heat sink parameter is numerically escalated, the temperature profile drops, but the temperature field increases when the heat source value is upsurged. Adding a substantial quantity of heat energy to the hybrid nanofluid during this operation raises the temperature field in the boundary layer region close to the shrinking sheet. Figure 9 describes the effect of the thermal radiation parameter (*Nr)* on the temperature profile. It can be seen that the temperature field declines in the region 0 ≤ *η* < 2.25 and it increases in the region 2.25 < *η* ≤ 4.5 as *Nr* is raised. Physically, the radiative component accelerates the motion of small particles, causing random migrating particles to collide and the resulting frictional energy to be converted to thermal energy. Ordinary nanofluids have a little lower temperature than hybrid nanofluids. Figure 10 illustrates the impact of the temperature ratio parameter $\left( {\theta_{w} } \right)$ on the temperature field. It can be observed that when $\theta_{w}$ is increased then there is a notable increase in the temperature profile. A linear thermal radiation phenomenon is achieved by considering $\theta_{w} = 1.$ Physically, the higher $\theta_{w}$ implies a remarkable difference between the wall and ambient temperature. The thermal boundary layer thickness improves as a result of the temperature change. The temperature of a hybrid nanofluid is higher than that of a regular nanofluid.

**Figure 11:**
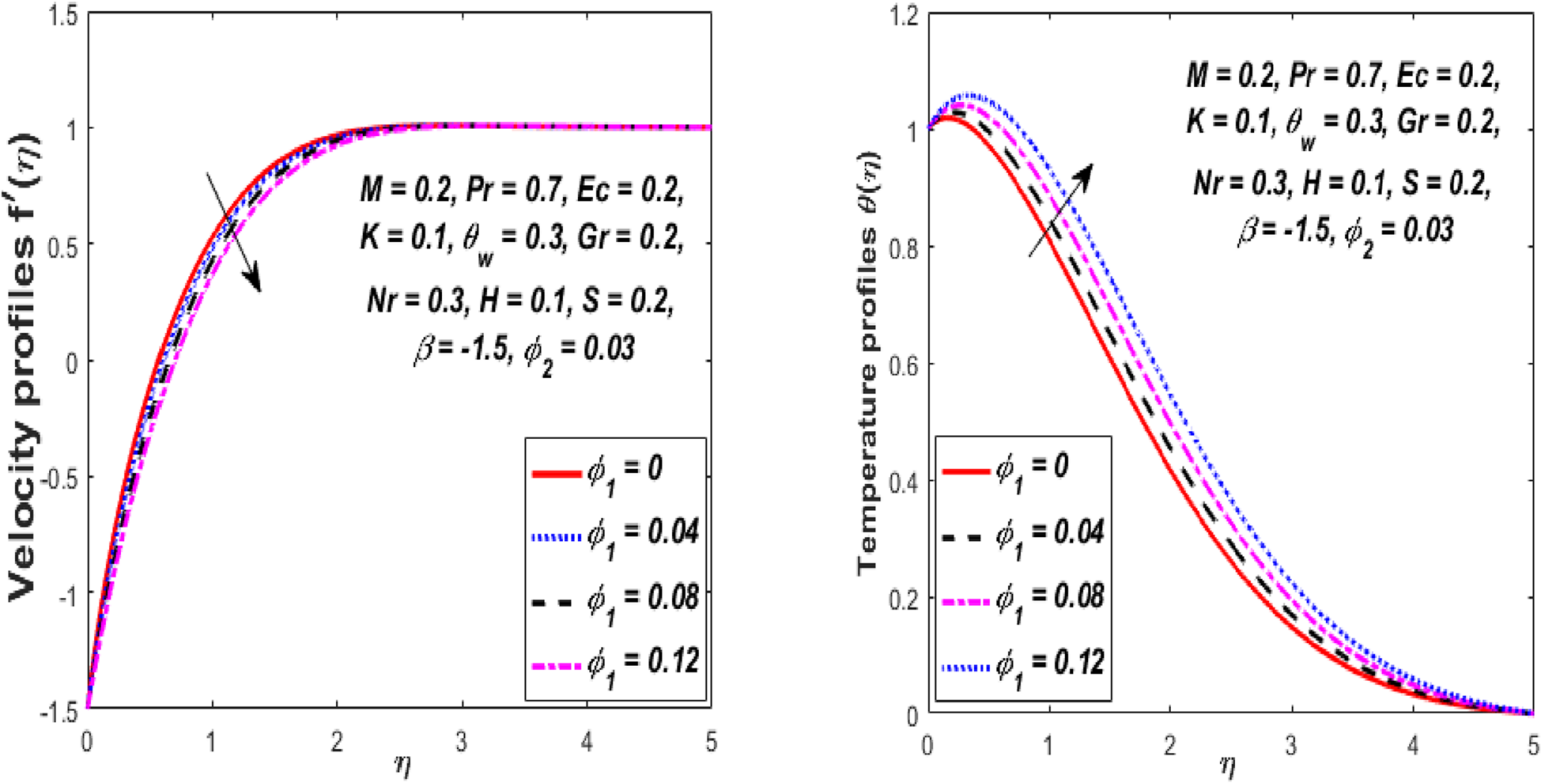
Influence of $\phi_{1}$ on velocity $f^{\prime}\left( \eta \right)$ and temperature $\theta \left( \eta \right)$ profiles.

**Figure 12:**
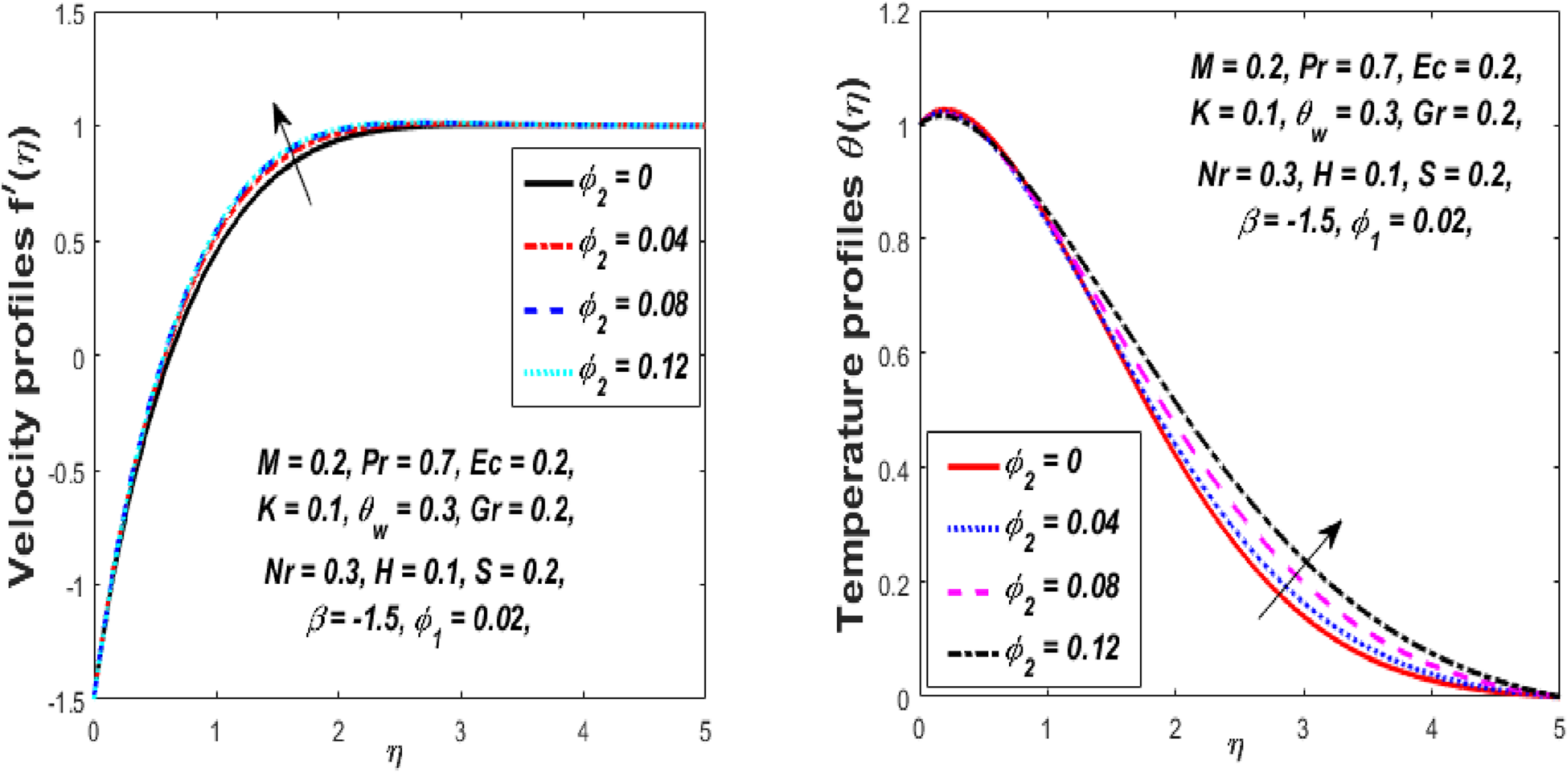
Influence of $\phi_{2}$ on velocity $f^{\prime}\left( \eta \right)$ and temperature $\theta \left( \eta \right)$ profiles.

The impression of the volume fraction of nanoparticles $\left( {\phi_{1} } \right)$ on the velocity and temperature fields is portrayed in Fig. 11. When $\phi_{1}$ increases, it can be noted that there is a fall in the velocity field while the temperature field upsurges for the hybrid nanofluid. Physically, for the higher volume fraction $\phi_{1}$, the momentum and thermal boundary layer become denser owing to the presence of (${\text{Al}}_{{2}} {\text{O}}_{{3}} {\text{ + Cu}}$) hybrid nanoparticles into the customary fluid which produces more resistance, and as a resultant, the velocity declines, and hence the temperature of the fluid escalates. Figure 12 shows the result of the nanoparticles volume fraction *(Cu) *$\phi_{2}$ on the velocity and temperature fields. It is detected that when $\phi_{2}$ is increased, the velocity and temperature fields augmented gradually. Physically, the density of hybrid nanofluid decreases owing to the higher values of $\phi_{2} ,$ which thus enhances the velocity and temperature. Accordingly, the intermolecular forces between the particles of hybrid nanofluids become weaker, and consequently, the hybrid nanofluid velocity accelerates.

Now, we discuss the comparison of nanofluid and hybrid nanofluid through the triangular MF. The volume fraction of nanoparticles $\phi_{1}$ and $\phi_{2}$ are considered to be TFNs (see Table 2) to build up the proposed problem using $\gamma {\text{ - cut}}$ approach $\left( {0 \le \gamma \le 1} \right)$. The temperature (Eq. 14) and velocity (Eq. 17) equations are assumed to be FDEs. These are converted into lower and upper bounds with the help of $\gamma {\text{ - cut}}$ technique and solved by employing the numerical technique bvp4c.

**Figure 13:**
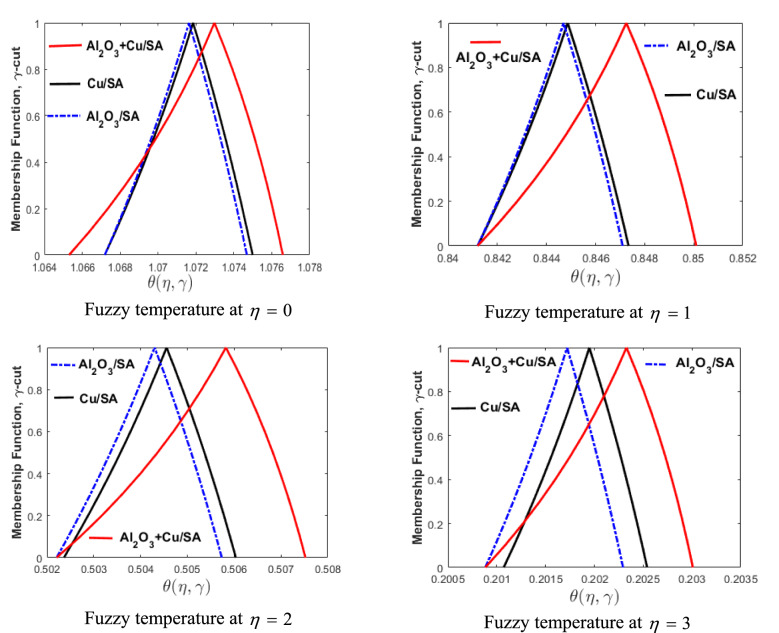
Comparison of ${\text{Al}}_{{2}} {\text{O}}_{{3}} {\text{/SA,}}$ ${\text{Cu/SA}}$ and ${\text{Al}}_{{2}} {\text{O}}_{{3}} {\text{ + Cu/SA}}$ hybrid nanofluid for varying of $\eta .$

**Figure 14:**
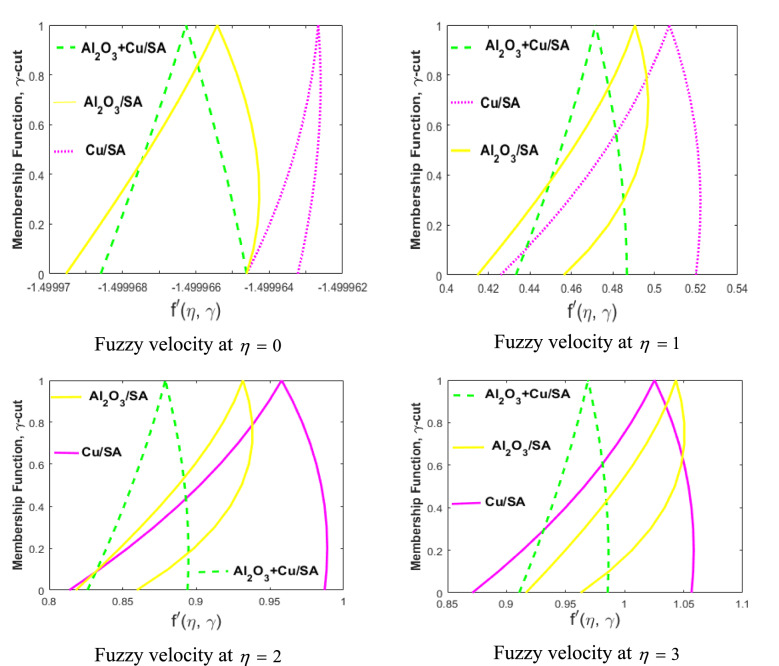
Comparison of ${\text{Al}}_{{2}} {\text{O}}_{{3}} {\text{/SA,}}$ ${\text{Cu/SA}}$ and ${\text{Al}}_{{2}} {\text{O}}_{{3}} {\text{ + Cu/SA}}$ hybrid nanofluid for varying of $\eta .$

Figure 13 represents the comparison of nanofluids ${\text{Al}}_{{2}} {\text{O}}_{{3}} {\text{/SA}}$ $\left( {\phi_{1} } \right),$ ${\text{Cu/SA}}$ $\left( {\phi_{2} } \right),$ and ${\text{Al}}_{{2}} {\text{O}}_{{3}} {\text{ + Cu/SA}}$ hybrid nanofluid through the MFs of fuzzy temperature profile for different values of $\eta .$ In these figures, we have examined three different cases. Blue-dashed lines represent the case when $\phi_{1}$ is taken as TFN and $\phi_{2} = 0$. Black lines show the variation of $\phi_{2}$ whereas $\phi_{1} = 0$. In the third case, hybrid nanofluid is reflected with both $\phi_{1}$ and $\phi_{2}$ non-zero. Also, the horizontal axis shows the fuzzy temperature profile for varying $\eta ,$ while the vertical axis shows the membership values of fuzzy temperature profile for varying $\gamma {\text{ - cut}}{.}$ It is observed that the hybrid nanofluid ${\text{Al}}_{{2}} {\text{O}}_{{3}} {\text{ + Cu/SA}}$ is better when compared with nanofluid ${\text{Al}}_{{2}} {\text{O}}_{{3}} {\text{/SA}}$ or ${\text{Cu/SA}}{.}$ As the temperature difference in the case of hybrid nanofluid is more prominent than the other two. The collective thermal conductivities of ${\text{Al}}_{{2}} {\text{O}}_{{3}}$ and ${\text{Cu}}$ are added in a hybrid nanofluid that allows passing the maximum heat transfer rate. ${\text{Cu/SA}}$ shows superior heat transfer rate when compared with ${\text{Al}}_{{2}} {\text{O}}_{{3}} {\text{/SA}}$ nanofluid as the thermal conductivity of *Cu* is greater than ${\text{Al}}_{{2}} {\text{O}}_{{3}} .$ The same three cases (of Fig. 13) are discussed in Fig. 14 for the fuzzy velocity profile against $\eta .$ Also, it has been observed that the fuzzy velocity of ${\text{Cu/SA}}$ nanofluid is maximum as compared to ${\text{Al}}_{{2}} {\text{O}}_{{3}} {\text{/SA}}$ or hybrid nanofluid.

The outcome of numerical values of several physical parameters on skin-friction coefficient and Nusselt number of hybrids nanofluid are organized in Table 4. It may be perceived that skin friction is enhanced over the surface of a permeable shrinking/stretching sheet for the larger numerical values of rate of mass transfer, heat source or sink parameter, Prandtl number, second-grade fluid parameter, magnetic parameter, the volume fraction of hybrid nanoparticles $\phi_{1}$ and $\phi_{2}$ while it reduced for the higher values velocity ratio parameter, buoyancy ratio parameter, Eckert number, thermal radiation parameter, and temperature ratio parameter. Also, if more hybrid nanoparticles are added, then the skin friction gets heightened. When an orthogonal magnetic field is applied to a hybrid nanofluid flowing over a sheet, the metallic nanoparticles are dragged, which raises the skin fraction. The next column of Table 4 exhibits the effect of the same engineering parameters on the $Nu_{x}$ (heat transfer rate over the surface of a permeable shrinking/stretching sheet). When the velocity ratio parameter, rate of mass transfer, buoyancy ratio parameter, thermal radiation parameter, heat source or sink parameter, Prandtl number, $\phi_{1}$ and $\phi_{2}$ are enhanced, then the rate of heat transfer improves whereas, it declines for the larger values of the temperature ratio parameter, magnetic parameter, second-grade fluid parameter, and Eckert number. Furthermore, when compared to regular fluids, the hybrid nanofluids are upsurge the heat transfer rate.

**Table 4:** Numerical values of skin-friction coefficient (*f* ″(0)) and Nusselt number (*θ*′(0)) for different values of control parameters.

$\beta$	*K*	*Gr*	*Ec*	*Pr*	*M*	*Nr*	$\theta_{w}$	*H*	$\phi_{1}$	$\phi_{2}$	s	$- R_{{e_{x} }}^{\frac{1}{2}} C_{fx}$	$R_{{e_{x} }}^{{\frac{ - 1}{2}}} Nu_{x}$
1.1	0.1	0.3	0.1	0.7	0.2	0.2	1.1	0.1	0.02	0.02	0.1	0.0777193	1.0581736
1.2	–	–	–	–	–	–	–	–	–	–	–	0.3285653	1.0679864
1.3	–	–	–	–	–	–	–	–	–	–	–	0.5932094	1.0757930
–	0	–	–	–	–	–	–	–	–	–	–	0.0655866	0.8414775
–	0.1	–	–	–	–	–	–	–	–	–	–	0.0716423	0.8414398
–	0.15	–	–	–	–	–	–	–	–	–	–	0.0746716	0.8414209
–	–	0.1	–	–	–	–	–	–	–	–	–	0.1801700	0.8223483
–	–	0.2	–	–	–	–	–	–	–	–	–	0.0661369	0.8287223
–	–	0.3	–	–	–	–	–	–	–	–	–	0.0096727	0.8317369
–	–	–	0.1	–	–	–	–	–	–	–	–	0.1801700	0.8223483
–	–	–	0.3	–	–	–	–	–	–	–	–	0.1801579	0.8211763
–	–	–	0.5	–	–	–	–	–	–	–	–	0.1801459	0.8200045
–	–	–	–	0.1	–	–	–	–	–	–	–	0.0115632	0.3430460
–	–	–	–	0.4	–	–	–	–	–	–	–	0.0527912	0.6839234
–	–	–	–	0.9	–	–	–	–	–	–	–	0.0765798	0.9601067
–	–	–	–	–	0.2	–	–	–	–	–	–	0.0661369	0.8287223
–	–	–	–	–	0.6	–	–	–	–	–	–	0.0866545	0.8276831
–	–	–	–	–	1	–	–	–	–	–	–	0.1056417	0.8267557
–	–	–	–	–	–	0.1	–	–	–		–	0.0701105	0.8152293
–	–	–	–	–	–	0.5	–	–	–	–	–	0.0563071	0.8725772
–	–	–	–	–	–	0.9	–	–	–	–	–	0.0465546	0.9339197
–	–	–	–	–	–	–	1.1	–	–	–	–	0.0777193	1.0581736
–	–	–	–	–	–	–	1.2	–	–	–	–	0.0759181	1.0159423
–	–	–	–	–	–	–	1.3	–	–	–	–	0.0739262	0.9706931
–	–	–	–	–	–	–	–	0	–	–	–	0.0687519	0.8784832
–	–	–	–	–	–	–	–	0.2	–	–	–	0.0735019	0.9718093
–	–	–	–	–	–	–	–	0.4	–	–	–	0.0777193	1.0581736
–	–	–	–	–	–	–	–	–	0.03	–	–	0.0681963	0.8331914
–	–	–	–	–	–	–	–	–	0.04	–	–	0.0703678	0.8376262
–	–	–	–	–	–	–	–	–	0.05	–	–	0.0726554	0.8420298
–	–	–	–	–	–	–	–	–	–	0.01	–	0.0678193	0.8330017
–	–	–	–	–	–	–	–	–	–	0.03	–	0.0696515	0.8372399
–	–	–	–	–	–	–	–	–	–	0.05	–	0.0716423	0.8414398
–	–	–	–	–	–	–	–	–	–	–	0.1	0.0516429	0.7424399
–	–	–	–	–	–	–	–	–	–	–	0.3	0.0661369	0.8287223
–	–	–	–	–	–	–	–	–	–	–	0.5	0.08197858	0.9185217

## Conclusions

In this work, theistagnation-pointiflow of aisecond-gradeihybridinanofluid (${\text{Al}}_{{2}} {\text{O}}_{{3}} {\text{ + Cu/SA}}$) through a convectively heated permeable extending or shrinking sheet with heat sink or source, viscousidissipation, and nonlinear thermal radiation is studied. A numerical scheme bvp4c assists us to achieve the solution of the dimensionless mathematical equations. The effect of involved control parametersion velocityiand temperatureiprofiles is represented through figures. The Nusseltinumber and the skin frictionicoefficient are numerically expressed in a tabular form. The volumeifraction of ${\text{Al}}_{{2}} {\text{O}}_{{3}}$ and ${\text{Cu}}$ nanoparticles are taken as TFNs and discussed. The nonlinear coupled ODEs are converted into FDEs and then numerically solved by using the bvp4c scheme. For the authentication, present work is in good agreement as compared to existing work. A comparison of nanofluid with hybrid nanofluid through the triangular fuzzy membership functions is discussed. The important key outcomes are provided below.

- A rise in nanoparticle volume fraction $\phi_{1}$ and $\phi_{2}$ results in an increment in theithermal boundaryilayer and a decline in the velocity profile.
- The temperatureiprofile diminishes and velocityiprofile improves for the larger values of rate of massitransfer parameter, second-gradeifluidiparameter, and buoyancyiratio parameter.
- The heat transfer rate raises for higherivalues of nonlinearithermal radiationiparameter, temperatureiratio parameter, and Eckertinumber.
- When the heatisource or sinkiparameter is enhanced, the temperature profile is magnified, while when the heatisource orisinkiparameter is diminished, theitemperature profile reduces.
- Theiskinifrictionicoefficient enhances when heatisource or sinkiparameter, rate of mass transfer parameter, second-grade fluid parameter, $\phi_{1}$ and $\phi_{2}$ increases and declines when buoyancyiratioiparameter, thermaliradiationiparameter, velocity ratioiparameter, and temperature ratioiparameter increase.
- The Nusseltinumberienhances with growing values of thermal radiationiparameter, buoyancy ratio parameter, $\phi_{1}$ and $\phi_{2}$ while diminishes with risingivalues ofitemperature ratioiparameter, magnetic parameter, and second-grade fluid parameter.
- Through triangular fuzzy MFs, it is witnessed that the ${\text{Al}}_{{2}} {\text{O}}_{{3}} {\text{ + Cu/SA}}$ hybrid nanofluids are highly capable to boost the heat transfer rate as compared to ${\text{Al}}_{{2}} {\text{O}}_{{3}} {\text{/SA}}$ and ${\text{Cu/SA}}$ nanofluids.

The cited scientific contribution might aid in the advancement of extrusion processes, heat transfer enhancement, biotechnology, and nanotechnology. The simulations given in this paper may be expanded to three-dimensional flows with various flow properties such as nanofluids, activation energy, Joule heating, thermal radiation, and entropy formation. Furthermore, for such specified accelerated surfaces issues, a variety of numerical techniques can be used.

## List of symbols

$$x,\,\,y$$: Cartesian coordinates
$$u,\,\,v$$: Velocity components
$B_{0}$: Uniform Magnetic field
$g$: Acceleration due to gravity
$\alpha_{2}$: Second grade fluid parameter
$$T$$: Temperature
$T_{w} ,\,\,\,T_{\infty }$: Reference and ambient temperature
$Q_{0}$: Heat absorption/generation coefficient
$q_{r}$: Heat source of radiativity
$\rho_{hnf}$: Density of hybrid nanofluid
$\rho_{f}$: Density of fluid
$\mu_{hnf}$: Dynamic viscosity of hybrid nanofluid
$\mu_{f}$: Dynamic viscosity of the fluid
$$\eta$$: Similarity variable
$$\psi$$: Stream function
$\beta$: Shrinking/stretching rate parameter
$$M$$: Magnetic parameter
$\theta_{w}$: Temperature ratio parameter
$$Ec$$: Eckert number
$K$: Dimensionless Second-grade fluid parameter
$$\Pr$$: Prandtl number
$f^{\prime}(\eta ,\,\gamma )$: Fuzzy velocity profile
$$Nu_{x}$$: Nusselt number
${\text{Re}}_{x}$: Local Reynold number
$\sigma_{hnf}$: Electrical conductivity of hybrid nanofluid
$\sigma_{f}$: Electrical conductivity of the fluid
$\alpha_{hnf}$: Thermal diffusivity of hybrid nanofluid
$\left( {\beta_{T} } \right)_{hnf}$: Thermal expansion coefficient of hybrid nanofluid
$H$: Dimensionless heat absorption/generation coefficient
$Nr$: Thermal radiation parameter
*s*: Rate of mass transfer parameter
$f\left( \eta \right)$: Normal component of the flow
$$\theta \left( \eta \right)$$: Dimensionless temperature
$s_{1}$: Solid nanoparticles of $Al_{2} O_{3}$
$s_{2}$: Solid nanoparticles of $Cu$
$$Gr$$: Grashof number
$\left( {\rho c_{p} } \right)_{hnf}$: Heat capacity of hybrid nanofluid
$\left( {\rho c_{p} } \right)_{f}$: Heat capacity of hybrid flui
$k_{hnf}$: Thermal conductivity of the hybrid nanofluid
$k_{f}$: Thermal conductivity of the hybrid fluid
$\phi_{1}$: Volume fraction of alumina
$\phi_{2}$: Volume fraction of copper
$\nu_{f}$: Kinematic viscosity of fluid
$\nu_{hnf}$: Kinematic viscosity of hybrid nanofluid
$\gamma$: Level or cut technique
$$\mu_{{\overline{\Omega }}} (\eta )$$: Membership function
$\overline{\theta }(\eta ,\,\gamma )$: Fuzzy temperature profile
$Cf_{x}$: Skin-friction coefficient
